# Geographic distribution of methyltransferases of *Helicobacter pylori*: evidence of human host population isolation and migration

**DOI:** 10.1186/1471-2180-9-193

**Published:** 2009-09-08

**Authors:** Filipa F Vale, Francis Mégraud, Jorge MB Vítor

**Affiliations:** 1 Engineering Faculty, Portuguese Catholic University, Estrada Octávio Pato, 2635-631 Rio de Mouro, Portugal; 2 INSERM U853, F 33076, Bordeaux, France; 3 iMed.UL (MedChem Division), Faculty of Pharmacy, University of Lisbon, Av. das Forças Armadas, 1649-003 Lisboa, Portugal

## Abstract

**Background:**

*Helicobacter pylori *colonizes the human stomach and is associated with gastritis, peptic ulcer, and gastric cancer. This ubiquitous association between *H. pylori *and humans is thought to be present since the origin of modern humans. The *H. pylori *genome encodes for an exceptional number of restriction and modifications (R-M) systems. To evaluate if R-M systems are an adequate tool to determine the geographic distribution of *H. pylori *strains, we typed 221 strains from Africa, America, Asia, and Europe, and evaluated the expression of different 29 methyltransferases.

**Results:**

Independence tests and logistic regression models revealed that ten R-M systems correlate with geographical localization. The distribution pattern of these methyltransferases may have been originated by co-divergence of regional *H. pylori *after its human host migrated out of Africa. The expression of specific methyltransferases in the *H. pylori *population may also reflect the genetic and cultural background of its human host. Methyltransferases common to all strains, M. HhaI and M. NaeI, are likely conserved in *H. pylori*, and may have been present in the bacteria genome since the human diaspora out of Africa.

**Conclusion:**

This study indicates that some methyltransferases are useful geomarkers, which allow discrimination of bacterial populations, and that can be added to our tools to investigate human migrations.

## Background

*Helicobacter pylori *colonizes about half of the human population and is associated with several gastrointestinal diseases, such as gastritis, peptic ulcer, and gastric cancer [1,2]. The similar pattern of human and *H. pylori *geographic diversity and distribution suggests a co-evolution between bacteria and man, which can be used to understand human migrations [2]. The *H. pylori *distribution pattern follows the human migration roots, which suggests that the colonization of the human stomach occurred before modern man left East Africa [2-5].

Several *H. pylori *gene alleles present different prevalence rates among the world *H. pylori *population. This is the case for *vacA *that presents allelic diversity of the s-, m- and i-region [6,7]. The *cag*A gene DNA motifs also presents a clear geographic association, and five types of deletion, insertion, and substitution motifs were found at the 3' end of the *H. pylori cag *pathogenicity island associated with different human populations [8]. Another study confirms that the candidate virulence factors, *vacA*, *cagA *and *iceA*, cluster according to geographic region [9]. Interestingly, *iceA *has two known alleles, *iceA1 *and *iceA2 *[10,11], with the locus *iceA1 *encoding a protein with 52% identity with the restriction endonuclease NlaIII [12]. Likewise, the *rpoB *gene, which codes for RNA polymerase β subunit, presents allelic diversity between Asian and non-Asian strains at the amino acid threonine, which is present only in Asian strains (two thirds of the Asian strains), while it is substituted with alanine in strains of western origin [13]. Allelic diversity according to the geographic distribution was also found for the *babA *and *babB *genes, which code for outer membrane proteins [14,15].

The transposable element ISHp60 presents a non-random geographic distribution, being more frequent in Latin America and rarer in East Asia [16]. The *hopQ *(omp27) alleles show high genetic variability, and type I alleles from Western and Asian *H. pylori *strains were similar and markedly different from type II *hopQ*. Type II *hopQ *alleles were frequently identified in Western *H. pylori *strains, but rarely in East Asian strains [17].

One class of highly variable genes in the *H. pylori *genome is the restriction and modification (R-M) systems [18]. R-M systems usually comprise both a restriction endonuclease (REase) that recognizes a specific DNA sequence and cuts both strands and a cognate DNA methyltransferase (MTase) that methylates the same DNA sequence, thus protecting it from being cleaved by the companion REase [19]. The sequenced *H. pylori *strains, strain 26695 [20], strain J99 [18], strain HPAG1 [21], and strain G27 [22], revealed 26 putative restriction and modification (R-M) systems in the first two strains, and 31 and 34 in the last two [23]. Only a reduced number of the expressed MTases in strains J99 and 26695 are common [24,25]. A small fraction of the potential type II R-M systems in strains J99 and 26695 appear to be fully functional, but different sets of these R-M genes are functionally active in each strain [26,27]. The analysis of the expression of MTases in other strains confirmed the high number of expressed enzymes, as well as their diversity among strains [27-31]. Likewise, non-pylori *Helicobacter *spp. appears to express a high number of MTases, as it was previously determined for *H. pylori *[32]. It has been proposed that the diversity of R-M systems in *H. pylori *is high enough to be used as a typing method [30,31]. Takata *et al*. studied the genomic methylation status in 122 *H. pylori *strains from several world regions, by performing hydrolysis with 14 REases. This study confirmed the diversity and the high number of expressed MTases, but did not reveal any significant association with the *H. pylori *geographic origin [29].

The biological function of R-M systems has yet to be ascertained. Typically, R-M systems function like an immune system to protect bacteria against invasion of foreign DNA, especially of bacteriophages [33]. However there is a limited number of reports on *H. pylori *phages [34-36], which also support other biological roles for R-M systems. These may include regulation of genetic exchange in the naturally competent *H. pylori *[37,38] or promotion of homologous recombination between DNA fragments produced by incomplete REase digestion [39]. The linkage of R-M genes allows for simultaneous loss of R and M genes, while physical separation of their gene products permits the hydrolysis of the genomic DNA by the residual REase present in daughter cells, leading to postsegregational killing. This occurs because when cells divide, the daughter cells lose the ability to protectively methylate all recognition sites in the newly synthesized chromosome, causing the cleavage of unmethylated sites by the residual REase still present in the bacterial cytoplasm [40,41]. The stability of the expression appears to be rather stable (94.9%) in strains isolated from the same patient at different times [30].

In the present study, the majority of strain specific genes with known function, e. g., those that code for restriction and modification (R-M) systems [18], were evaluated for their association with the geographic origin of the *H. pylori *strains. Since *H. pylori *co-evolved with man [2], it is important to understand if these strain specific genes (restriction and modification genes) reflect a similar geographic distribution between man and bacteria characteristic of isolated population. The expression of 29 MTases was assessed by hydrolysis of genomic DNA with the cognate REases in 221 *H. pylori *strains from Africa, America, Asia and Europe. Data were statistically analysed using independence tests as well as multiple and multinomial logistic regression models. Here, we present a geographic pattern for 10 MTases expressed in *H. pylori *and two conserved MTases expressed in all strains tested. We further explored the association of these MTases with geographic clusters of *H. pylori *populations to determine if the divergence of regional *H. pylori *populations is associated with its human host migrations and genetic/cultural habits.

## Results

### DNA modification in strains from different geographic origin

The percentage of strains resistant to hydrolysis was determined after clustering the strains by country and continent. The total data set corresponds to 6409 DNA hydrolyses (221 × 29 REases, Additional file 1: Table S1). Analyses were done in duplicate for 250 of these digestions, with similar results (data not shown). All strains presented variable resistance to digestion, as previously described [18,24,25,27,30,31]. The resistance to hydrolysis ranged from 100%, with NaeI and HhaI, to 0% with Sau3AI (Additional file 2: Table S2 and Figure S1). The number of expressed MTases in *H. pylori *strains was high, as reported [18,26,27,29,30], with a total average of 15.8 ± 2.2, (range 9-20), among 27 tested REases (isoschizomers excluded).

### Selection of methyltransferases with non-random geographic distribution

A chi-square independence test was used to select the independent variables to be applied in the logistic regression models (Additional file 2: Table S3). Ten MTases were associated with the geographic origin of the strains analysed. A significant result was determined by the analysis of standardized residuals (std. residual) for all MTases presenting a geographic association, except M. MspI and M. TaqI (Table 1). A Fischer test was applied and all significant associations were confirmed (Additional file 2: Table S4).

**Table 1:** MTases presenting a statistical significant association with isolates of distinct geographic origin (Chi-square test).

MTase	Recognition sequence *	Chi-square	higher	smaller	Std.
				
		(p value)	expression in isolates from		Residual
M. AseI	ATTAAT	0.031	--	Africa	2.13
M. FokI	GGATG	0.001	America Asia	--	2.77 2.55
M. MspI	CCGG	0.036	--	--	
M. Hpy188I	TCNGA	0.002	America	--	2.05
M. Hpy99I	CGWCG	0.025	America	--	2.29
M. HpyCH4III	CANGT	<0.001	Africa America	--	-1.99 -2.21
M. DraI	TTTAAA	<0.001	Asia	--	5.36
M. BstUI	CGCG	0.006	Asia	--	2.81
M. FauI	CCCGC	0.004	Asia	--	-2.04
M. TaqI	TCGA	0.044	--	--	

* data from REBASE [23].

### Multiple logistic regression

The 10 MTases with significant association with strain origin (Table 1) were used as independent variables for the multiple logistic regression. A logistic regression was calculated to predict the strain origin (Europe versus non-Europe; or Africa versus non-Africa). Considering that the majority of strains are of European origin, the output variable, or dependent variable, was established as Europe/non-Europe. The model was statistically significant (p = 0.00040), i.e. the selected independent variables were significant for the output. Four MTases yielded significant results for the logistic regression model: M. AseI, M. FokI, M. MspI, and M. HpyCH4III. M. AseI expression is associated with the European group and the other 3 MTases with the non-European group (Additional file 2: Table S5). When the dependent variable is Africa/non-Africa origin and we use the same 10 independent variables, the full model is once again significant (p = 0.0001) (Additional file 2: Table S6). For this model we identified 5 significant MTases: M. AseI, M. MspI, M. Hpy188I, M. Hpy99I, and M. HpyCH4III. There was an association of the expression of M. MspI and M. HpyCH4II with African strains (Odds Ratio, OR>1). The other MTases were associated with the strains of non-African origin (OR<1).

### Multinomial logistic regression

A multinomial logistic regression presented a nominal outcome variable with 4 levels: Africa, Asia, America, and Europe. When the same 10 MTases (Table 1) were analysed the standard errors were greater than 2 for M. FokI and M. FauI (data not shown), which limited the interpretation of the model. Thus, the multinomial logistic regression was run again with 8 independent variables, although the other two MTases were significant to the full model (p < 0.05).

The multinomial logistic regression model revealed the absence of expression of M. MspI and M. HpyCH4III in the European group with OR = 4.51, and OR = 4.34, respectively. This strongly suggests that the expression of both MTases were more likely to be present in the African group than in the European group (Additional file 2: Table S7). Regarding the American and African groups, the expression of M. Hpy188I and M. Hpy99I was more likely to occur in the American group than in the African reference group, with OR = 0.17 and OR = 0.16, respectively. Concerning the Asian group, M. HpyCH4III was more frequent in the African group than in the Asian one, with OR = 16.98. M. BstUI was more likely to be present in the Asian group, with OR = 0.07. When the reference category corresponded to European isolates, the comparison with the African group yielded similar findings to the ones described previously, but allowed for the comparison between Europe and America, and Europe and Asia. Resistance to restriction by Hpy188I, Hpy99I and HpyCH4III was more likely to be observed in the American group than in the reference group, with OR values of 0.37, 0.35, and 0.19, respectively. The reference category and the Asian group assessment revealed an OR = 0.12 for M. BstUI, and an OR = 0.07 for M. DraI, which indicated that both MTases were more common among Asian strains (Additional file 2: Table S8).

A summary of the MTase geographic pattern determined by all statistical tests can be found in Table 2.

**Table 2:** List of MTases with statistically significant association with geographic area of strain isolation.

MTase	Expression*	Absence of expression*
M. AseI	**Europe** OR = 2.33; 95%CI (1.00-5.46) ^a)^	**Africa** P-value = 0.03083 *Std. Residual *2.13^e)^ OR = 0.27; 95%CI (0.10-0.75) ^b)^
M. BstUI	**Asia** P-value = 0.00639 *Std. Residual *2.81^e)^ OR = 1/0.12 = 8.33; 95%CI (1.37-50.00) ^c)^ OR = 1/0.07 = 14.29; 95%CI (2.13-100.00) ^d)^	**Africa** OR = 0.07; 95%CI (0.01-0.47) ^d)^ **Europe** OR = 0.12; 95%CI (0.02-0.73) ^c)^
M. DraI	**Asia** P-value < 0.00001 *Std. Residual *5.36^e)^ OR = 1/0.07 = 14.29; 95%CI (2.63-100.00) ^c)^	**Africa** **Europe** OR = 0.07; 95%CI (0.01-0.38) ^c)^
M. FauI	**Asia** P-value = 0.00403 *Std. Residual *-2.04^e)^	
M. FokI	**America** P-value = 0.00058 *Std. Residual *2.77^e)^ **Asia** P-value = 0.00058 *Std. Residual *2.50^e)^	**Africa** **Europe** OR = 0.12; 95%CI (0.02-0.70) ^a)^
M. Hpy188I	**America** P-value = 0.00177*Std. Residual *2.05^e)^ OR = 1/0.17 = 5.88; 95%CI (1.89-20.00) ^d)^ OR = 1/0.37 = 2.70; 95%CI (1.09-6.67) ^c)^ **Asia**	**Africa** OR = 0.35; 95%CI (0.14-0.87) ^b)^ OR = 0.17; 95%CI (0.05-0.53) ^d)^ **Europe** OR = 0.37; 95%CI (0.15-0.92) ^c)^
M. Hpy99I	**America** P-value = 0.02544 *Std. Residual *2.29^e)^ OR = 1/0.16 = 6.25; 95%CI (1.79-20.00) ^d)^ OR = 1/0.35 = 2.86; 95%CI (1.14-7.14) ^c)^	**Africa** OR = 0.35; 95%CI (0.12-0.99) ^b)^ OR = 0.16; 95%CI (0.05-0.56) ^d)^ **Europe** OR = 0.35; 95%CI (0.14-0.88) ^c)^
M. HpyCH4III	**America** P-value = 0.00015 *Std. Residual *-2.21^e)^ OR = 1/0.19 = 5.26; 95%CI (1.15-25.00) ^c)^ **Africa** P-value = 0.00015 *Std. Residual *-1.99^e)^ OR = 4.44; 95%CI (1.46-13.47) ^b)^ OR = 1/0.23 = 4.35; 95%CI (1.47-12.50) ^c)^ OR = 4.34; 95%CI (1.46-12.87) ^d)^ OR = 16.98; 95%CI (2.33-123.98) ^d)^	**Asia** OR = 1/16.98 = 0.06; 95%CI (0.01-0.43) ^d)^ **Europe** OR = 0.41; 95%CI (0.20-0.88) ^a)^ OR = 1/4.34 = 0.23; 95%CI (0.08-0.68) ^d)^ OR = 0.23; 95%CI (0.08-0.68) ^c)^ OR = 0.19; 95%CI (0.04-0.87) ^c)^
M. MspI	**Africa** P-value = 0.03638^e)^ OR = 4.42; 95%CI (1.46-13.43) ^b)^ OR = 1/0.22 = 4.55; 95%CI (1.49-14.29) ^c)^ OR = 4.51; 95%CI (1.49-13.67) ^d)^	**Europe** OR = 0.45; 95%CI (0.22-0.94) ^a)^ OR = 1/4.51 = 0.22; 95%CI (0.07-0.67) ^d)^ OR = 0.22; 95%CI (0.07-0.67) ^c)^

* Statistical analysis information:

a) Multiple logistic regression: dependent variable Europe or non-Europe;

b) Multiple logistic regression: dependent variable Africa or non-Africa;

c) Multinomial regression: reference category Europe;

d) Multinomial regression: reference category Africa;

e) Chi-square independence test (p-value and *std. residual*);

Note: in multinomial regression Odds Ratio (OR) values are determined for the absence of expression. The introduction of the inverse value allows the indication of OR value for presence of expression of each MTase. A OR 95% confidence interval is presented.

## Discussion

The considerable genetic diversity among strains of *H. pylori *[42] has already been used to discriminate between closely related human populations, that could not be discriminated by human genetic markers. *H. pylori *sequence analysis has the potential to distinguish short term genetic changes in human populations [43].

Most methyltransferases genes are part of restriction and modification systems in *H. pylori *genome [18,23,44]. These genes represent about 2% of the total number of genes [18,20,21], a very high proportion when compared with the mean percentage of methyltransferase (M) genes per sequenced genome in *Bacteria *(0.50%) [23]. The average number of R-M genes present in *H. pylori *sequenced genomes is 30, an extremely high value considering all sequenced bacterial genomes, with an average of 4.3 R-M systems per genome [23]. In addition to the high number of R-M systems present in *H. pylori *genome, which represent more than half of the strain-specific genes [45,46], these R-M systems also present a high diversity among strains [18,24,25,27-29,47], allowing them to be used as a typing system [30,31]. Moreover, some R-M systems are more prevalent in *H. pylori *than others, resulting in rare, medium, and frequent R-M systems [29,30,48]. The present study confirms the high number of M genes expressed per *H. pylori *strain, independently of its geographic origin and suggests that MTase expression is clearly associated with strain origin. To date, there is only one study [29] specifically designed to characterize MTases expression from different geographic origins. This report confirmed the diversity and the high number of expressed MTases, but did not reveal any significant MTase association with the geographic origin of *H. pylori *[29]. The difficulty in finding an association with geographic origin, may be due to the low number of strains analysed (122 strains),, which included only 3 strains from Africa as well as the limited number of MTases tested (14 REases). Table 2 summarizes MTases that present statistically significant geographic association. The odds ratio may present small differences for the same MTase, given analysis by several logistic regression models. Regardless, the values are always significant for an association between MTase and strain origin.

Our results suggest that the pattern of some *H. pylori *MTases is geographically defined, which may indicate that it is the result of geographic isolation of its human host or of the co-divergence of *H. pylori *MTases with host since the migration of modern human out of Africa. R-M systems present a lower G+C content than the total genome (Table 3), which has been considered as evidence for horizontal gene transfer [49-51]. Frequently, genes coding for R-M systems are within or adjacent to insertions with long target duplications, which suggests a similar transposon insertion with longer duplications, in agreement with an horizontal gene transfer [52]. Horizontal gene transfer of *H. pylori *MTases could favour the geographic isolation hypothesis. However, if we consider that phase variation does not seem to appear in R-M systems [53], and that temporal analysis of gene expression appears to be rather stable [30], MTases are likely not that mobile among genomes. Even though R-M systems may be mainly acquired by horizontal gene transfer, the fact that their expression appears to be stable after acquisition [30,53], arguing for a post segregational killing effect [41,54,55], and that *H. pylori *transmission occurs mainly within the same nuclear family or community [56-58], supports the concept of conservation of some R-M systems since the diaspora out of Africa [59], and the acquisition of other R-M genes later on, in specific geographic areas. Finally, the existence of MTases common to all geographic groups, M. NaeI and M. HhaI, is consistent with the hypothesis of *H. pylori *and *Homo sapiens *co-evolution after the human out-of-Africa movement [2,3]. It is assumed that modern humans appeared first in Africa, then in Asia, and from this continent they settled in three neighbouring regions: Oceania, Europe and America [4]. All *H. pylori *strains express the MTases M. HhaI and M. NaeI, suggesting that they have been present in the genome since the beginning of human dispersion from the Africa continent. Moreover, M. HhaI is an isoschizomer of M. Hpy99III, M. HpyORF1059P and M. HpyAVIII, which are MTases identified in *H. pylori *strains J99 (coded by *hpy99IIIM*), HPAG1 (coded by *hpyHORF1059MP*), 26695 (coded by *hpyAVIIIM*), and Shi470 (coded by *HPSH_05770*), respectively [23,60]. The identification of M. HhaI isoschizomers in three sequenced strains is in agreement with the hypothesis of these MTases being present in the *H. pylori *genome since the beginning of the human migrations.

**Table 3:** Genomes with higher number of predicted M genes [23].

Organism	Genome size (Mbp)	Total genes	M genes	**% M Genes **^a)^	**% GC Genome **^b)^	**% GC RM genes **^c)^
*Microcystis aeruginosa *NIES-843	5.84	6312	51	0.81	42	40
*Microcystis aeruginosa *PCC 7806	?	?	42	?	42	40
*Roseiflexus sp*. RS-1	5.80	4517	38	0.83	60	58
*Roseiflexus castenholzii *DSM 13941	5.72	4330	36	0.83	60	56
*Campylobacter upsaliensis *RM3195	1.77	1998	34	1.70	34	34
*Helicobacter pylori *G27	1.65	1493	34	2.28	38	37
*Helicobacter pylori *HPAG1	1.60	1536	32	2.08	39	37
*Helicobacter pylori *Shi470	1.61	1569	32	2.04	38	36
*Orientia tsutsugamushi *Boryong	2.13	1182	31	2.62	30	28
*Helicobacter acinonychis *Sheeba	1.55	1612	29	1.80	38	35
*Helicobacter pylori *P12	1.67	1567	29	1.85	38	36
*Cenarchaeum symbiosum*	2.05	2017	28	1.39	57	52
*Helicobacter pylori *26695	1.67	1576	28	1.78	39	36
*Helicobacter pylori *J99	1.64	1489	28	1.88	39	36

a) percentage of M genes (M genes/total genes)

b) percentage of GC content in the sequenced genome

c) mean percentage of GC content among R-M system genes present within the genome

**Figure 1:**
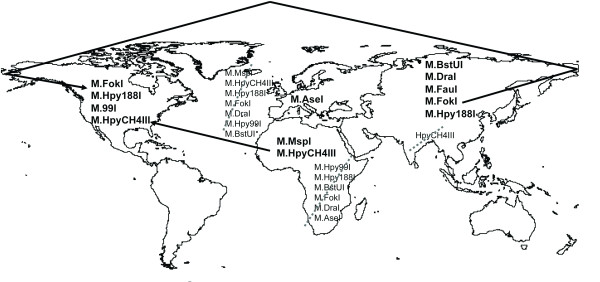
**Geographic distribution of *H. pylori *genomic methylation**. MTases with specific geographic origin are in bold. Arrows indicate MTases that are associated with a strain from more than a continent, according to human migrations predicted by Cavalli-Sforza. Grey dashed lines indicate MTases, whose absence is significantly associated with continent of strain origin.

It has been proposed that genes coding for R-M system were acquired recently, by horizontal gene transfer, with new systems being constantly acquired while old ones are inactivated or eliminated [27]. Our results support the hypothesis that at least some R-M systems were acquired since human migration out of Africa, while others were obtained later by geographically isolated bacterial populations. It is likely that the first MTases to be stably acquired by *H. pylori *genome were M. HhaI and M. NaeI, while the others were added later (Figure 1).

The other MTases showing a significant geographic association have probably been acquired at a later stage, depending on the *H. pylori *geographic localization. Thus, African strains are associated with M. HpyCH4III and M. MspI; Asian strains with M. BstUI, M. DraI, M. FauI, M. FokI and M. Hpy188I; European strains with M. AseI; and, finally, American strains with M. HpyCH4III, M. Hpy99I, M. Hpy188I e M. FokI (Figure 1).

Some MTases are common to more than one continent of origin, as is the case for M. FokI and M. Hpy188I, being both associated with Asia and America. Human migrations from Asia to America could provide some clues to this observation. The presence of both MTases supports the hypothesis of *H. pylori *and man co-evolution since the settlement of modern man (and *H. pylori*) in Asia, and posterior settlement in American land between 10,000 and 25,000 years ago, when a land bridge (the Bering Strait) connected Siberia and Alaska during the last ice age [4]. The expression of M. HpyCH4III in African and American strains, but not in the Asian ones, suggests that this MTase was acquired later and that the corresponding strains gained access to America in a second wave of migration of African slaves [4]. It should be pointed out that most of the American strains analysed are from South and Central American countries, which also experienced human traffic of African origin, although to a smaller extent when compared with other countries, such as Brazil. FokI's resistance to cleavage, which in the present study associated with Asian and American strains, has been previously reported for American and Asian strains, but not European and African [29], which is in agreement with the present study. Likewise, M. Hpy99I and M. Hpy188I associated with the American strains and were first isolated from the strains J99 and J188, also of American origin [18,61]. Only M. HpyCH4III, which is associated with American strains and not with Asian strains in the present study, was first isolated from a Chinese strain [31]. However, only strains from Singapore where considered in the Asian group, which is a limitation of this study. Nine out of ten of the Singapore strains (strains id. 33, 35-37, 39, 40, 42-43, available at *Helicobacter pylori *MLST Databases [62]) were common with the study of Falush et al. After the sequence of eight genes (including *vacA*, which presents allelic diversity) the majority of these strains were assigned to the hspEastAsia subpopulation [5]. This is in accordance with present results, since both studies clearly isolate these strains. For European, American and African strains, the dimension and diversity of these subgroups is higher, yielding more robust results.

It is thought that intra-familiar transmission plays a more important role in urban families than in rural families, where the horizontal transmission (for instance via non-parental caretakers) is predominant [63]. *H. pylori *appears to have established a long lasting colonization of its human host [64], probably being transmitted to subsequent human hosts by close human contact, e.g. between family or community members [63,65], depending on the genetic and social-cultural habits of each population. Our study supports previous reports on the co-evolution of *H. pylori *and man, in the sense that *H. pylori *reflects human migrations [3], being transmitted among individuals who have close contact with each other, but are not necessarily family members.

The biologic role of R-M systems is not completely understood. It remains an enigma if the increased number of MTases present in the *H. pylori *genome provides selective advantage to the bacteria, or if it is simply the result of the natural competence and postsegregational killing effects. Methylation has implications in gene expression [66], and *H. pylori *associates with several pathologies that may result from different sets of expressed genes [67]. For instance, DNA methylation by M. HpyAIV was shown to alter transcription of the catalase gene (katA) in *H. pylori *[68]. Further evidence is needed to understand if the high number and diversity of MTases expressed among *H. pylori *strains is beneficial for the bacteria and/or plays any role in pathogenicity.

## Conclusion

In conclusion, there is a clear association of some MTases with geographic groups of *H. pylori *strains, making them useful as geomarkers (Table 2). Indeed, other genes, as *cagA *or *vacA*, have allelic forms with particular geographic distributions [6,8,69]. Similar results are now observed for the majority strain-specific genes. M. HhaI and M. NaeI are common to all tested strains, which is consistent with the co-evolution theory of man and *H. pylori *[2,3] and suggests that their presence in bacterial genome preceded the human diaspora out of Africa. Finally, the association of MTases with geographical bacterial clusters may be observed in other bacterial species, and may reveal to be good geographic markers to trace bacterial evolution.

## Methods

### *H. pylori *strains

221 *H. pylori *strains were isolated from different regions (Africa, America, Asia and Europe) (Table 4). Strains belong to the collections of the *Helicobacter *and *Campylobacter *Reference Center of the Portuguese National Institute of Health (INSA), the *Helicobacter *and *Campylobacter *National Reference Center (Victor Segalen University, Bordeaux, France) and the Medical Microbiology Institute of Hannover (Germany). Strains belonging to INSA and *Helicobacter *and *Campylobacter *National Reference Center were randomly selected, except in cases in which all strains available for each sub-sample group were analysed (strains with African origin). DNA from *H. pylori *Singapore strains was randomly selected from East Asia *H. pylori *strain collection of the Medical Microbiology Institute of Hannover. Except for the country of origin, there is no further information about the ethnic group or ancestry of the human host providing the strain. Due to difficulty in obtaining strains from different geographic origins the number of strains from each continent is uneven.

**Table 4:** Geographic origin of *H. pylori *strains.

Continent	Country	Number of strains	Percentage
**Europe**		**146**	**66.1**
	Portugal	106	48.0
	France	11	5.0
	United Kingdom ^a)^	8	3.6
	Germany	6	2.7
	Sweden	9	4.1
	Norway	6	2.7
**Africa**		**38**	**17.2**
	Portuguese with African origin ^b)^	20	9.0
	Egypt	7	3.2
	Burkina Faso	11	5.0
**America**		**27**	**12.2**
	USA ^c)^	1	0.5
	Costa Rica	6	2.7
	Mexico	6	2.7
	Argentina	14	6.3
**Asia**		**10**	4.5
	Singapore	10	**4.5**
**Total**		221	100.0

a) Includes sequenced strain 26695;

b) Strains isolated from African patients with Portuguese residence were considered African;

c) Includes sequenced strain J99.

### *H. pylori *culture and genomic DNA extraction

*H. pylori *culture and DNA extraction were performed as previously described [30,70].

### Determination of MTase expression

Genomic DNA from each strain was hydrolysed with 29 REases: AciI, AseI, BseRI, BssHII, BssKI, BstUI, DdeI, DpnI, DpnII, DraI, EagI, FauI, Fnu4HI, FokI, HaeIII, HhaI, HpaII, Hpy188I, Hpy99I, HpyCH4III, HpyCH4IV, HpyCH4V, MspI, NaeI, NlaIII, Sau3AI, Sau96I, ScrFI and TaqI. Digestions were performed according to the manufacturer (New England Biolabs, USA), in a reaction volume of 50 μl. A negative control, without REase, was performed for a set of reactions. The results were coded on a binary matrix, where "0" indicates digestion observed (DNA is unmethylated), and "1" indicates no digestion, suggesting an active methyltransferase [30].

### Statistical analysis

For each MTase, a significance level of 0.05 was considered for the chi-square test, using the SPSS v.15.0. The chi-square test allowed to select MTases with an association with the origin of the isolates that were later used in logistic regression models. A Fischer test was performed to select MTases when the chi-square test was not valid (cells with expected counts lower than 5). The selected MTases were the independent variables (IV) in the logistic regression models. Multiple logistic regression used selected MTases as IV and a binary (dichotomous) dependent variable (DV) [71,72]. We chose Africa and non-Africa strains as DV, since it is accepted that *H. pylori *co-evolved with man since the human diaspora out of Africa [2-4]. Considering that the majority of strains were from European origin, another model was run, with European and non-European strains as DV. IV was also dichotomic (expression and no expression of MTase). The logistic regression allows for determination of the most adequate, parsimonious and biologically reasonable model describing the relation between the answer (or dependent variable) and the independent (or predictive) variable. Multinomial logistic regression models were also determined to analyze potential relationships between a non-metric dependent variable (four geographic origins) and metric or dichotomous independent variables and to compare multiple groups through a combination of binary logistic regressions [72]. The reference strain group was the African group, in agreement with the hypothesis of co-evolution of *H. pylori *and man [2,3] or the European group, since it consisted in a larger sub-sample.

## Authors' contributions

FV designed and performed research, analyzed data and prepared the manuscript. FM provided strain collection and contributed to the manuscript. JV designed research and contributed to the manuscript. All authors approved the final manuscript

## Supplementary Material

Additional file 1 **Vale et al. - Geographic distribution of methyltransferases of *Helicobacter pylori*: evidence of human host population isolation and migration - Global data matrix**. additional table presenting global results. Click here for file

Additional file 2 **Vale et al. - Geographic distribution of methyltransferases of *Helicobacter pylori*: evidence of human host population isolation and migration - Additional file of statistical analysis**. additional tables and figure presenting statistical analysis data. Click here for file
