# Supplementary material for: Geographic distribution of methyltransferases of Helicobacter pylori: evidence of human host population isolation and migration
Source: BMC Microbiol. 2009 Sep 8;9:193. doi: 10.1186/1471-2180-9-193 (PMC2749054; doi:10.1186/1471-2180-9-193)
Supplement: Additional file 2 — Vale et al. - Geographic distribution of methyltransferases of Helicobacter pylori: evidence of human host population isolation and migration - Additional file of statistical analysis. additional tables and figure presenting statistical analysis data. [file 1471-2180-9-193-S2.doc]

**Vale et al. – Geographic distribution of methyltransferases of *Helicobacter pylori*: evidence of human host population isolation and migration – Additional file of statistical analysis**


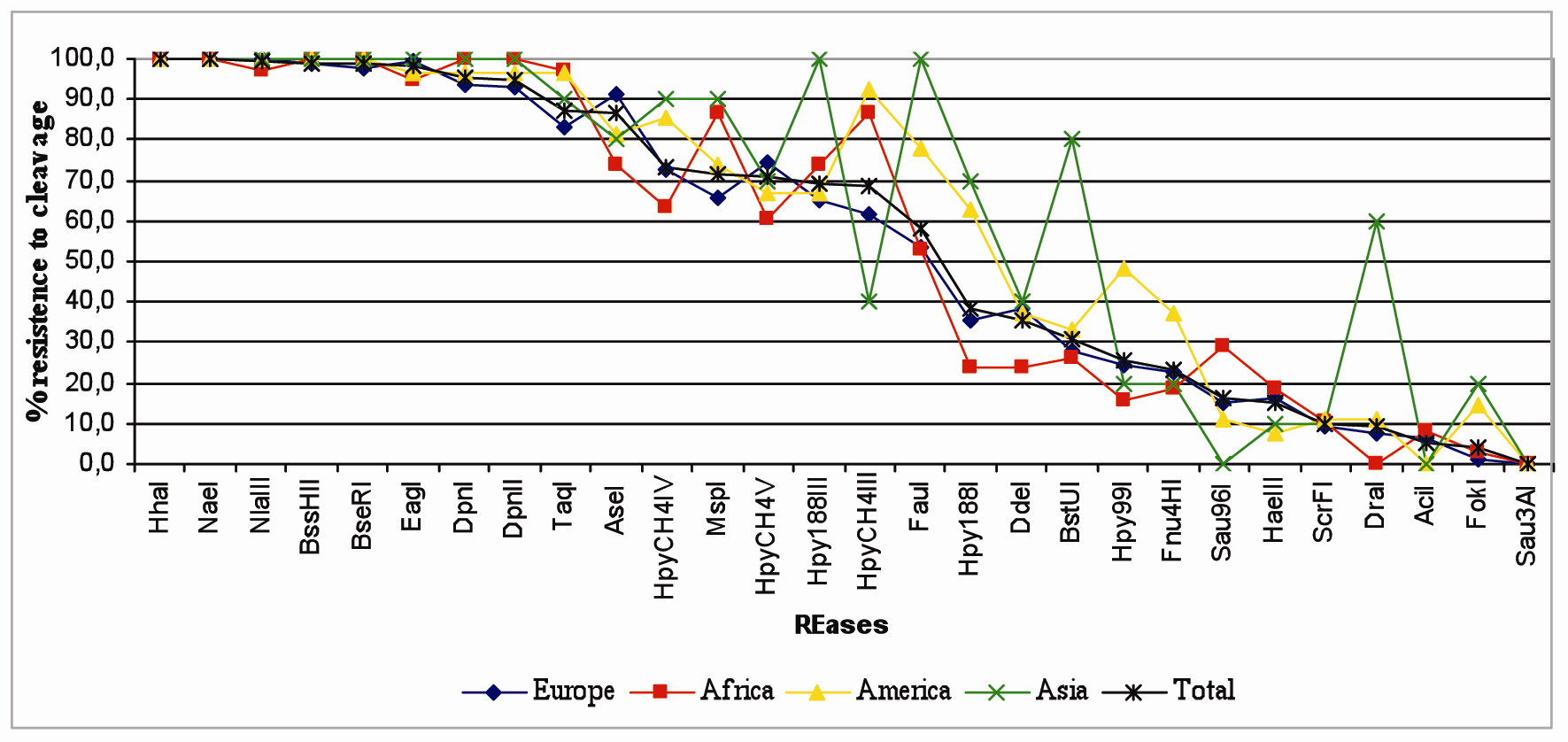


**Figure S1. Percentage of expression of all tested MTases by geographic origin.**

**Table S2. Percentage of resistance to cleavage according to strain’s continent origin.**

|  |  | Continent | | | | |  |
| --- | --- | --- | --- | --- | --- | --- | --- |
| REase | Recognition sequence and cut site* | Europe | Africa | America | Asia | Total |  |
| HhaI | GCG^C | 100.0 | 100.0 | 100.0 | 100.0 | 100.0 |  |
| NaeI | GCC^GGC | 100.0 | 100.0 | 100.0 | 100.0 | 100.0 |  |
| NlaIII | CATG^ | 100.0 | 97.4 | 100.0 | 100.0 | 99.5 | >75% |
| BssHII | G^CGCGC | 98.6 | 100.0 | 100.0 | 100.0 | 99.1 |
| BseRI | GAGGAG N10^ | 97.9 | 100.0 | 100.0 | 100.0 | 98.6 |
| EagI | C^GGCCG | 99.3 | 94.7 | 96.3 | 100.0 | 98.2 |
| DpnI | GA^TC | 93.8 | 100.0 | 96.3 | 100.0 | 95.5 |
| DpnII | ^GATC | 93.2 | 100.0 | 96.3 | 100.0 | 95.0 |
| TaqI | T^CGA | 82.9 | 97.4 | 96.3 | 90.0 | 87.3 |
| AseI | AT^TAAT | 91.1 | **73.7** | 81.5 | 80.0 | 86.4 |
|  |  |  |  |  |  |  |  |
| HpyCH4IV | A^CGT | 72.6 | 63.2 | **85.2** | **90.0** | 73.3 | >50%<75% |
| MspI | C^CGG | 65.8 | **86.8** | 74.1 | **90.0** | 71.5 |
| HpyCH4V | TG^CA | 74.7 | 60.5 | 66.7 | 70.0 | 71.0 |
| Hpy188III | TC^NNGA | 65.2 | 73.7 | 66.7 | **100.0** | 68.9 |
| HpyCH4III | CAN^GT | 61.6 | **86.8** | **92.6** | 40.0 | 68.8 |
| FauI | CCCGC N4^ | 53.4 | 52.6 | **77.8** | **100.0** | 58.4 |
|  |  |  |  |  |  |  |  |
| Hpy188I | TCN^GA | 35.6 | 23.7 | **63.0** | **70.0** | 38.5 | >25%<50% |
| DdeI | C^TNAG | 38.4 | 23.7 | 37.0 | 40.0 | 35.7 |
| BstUI | CG^CG | 28.1 | 26.3 | 33.3 | **80.0** | 30.8 |
| Hpy99I | CGWCG^ | 24.7 | **15.8** | 48.1 | 20.0 | 25.8 |
|  |  |  |  |  |  |  |  |
| Fnu4HI | GC^NGC | 22.6 | 18.4 | **37.0** | 20.0 | 23.5 | <25% |
| Sau96I | G^GNCC | 15.1 | **28.9** | 11.1 | 0.0 | 16.3 |
| HaeIII | GG^CC | 16.4 | 18.4 | 7.4 | 10.0 | 15.4 |
| ScrFI | CC^NGG | 9.6 | 10.5 | 11.1 | 10.0 | 10.0 |
| DraI | TTT^AAA | 7.5 | 0.0 | 11.1 | **60.0** | 9.0 |
| AciI | C^CGC | 6.2 | 7.9 | 0.0 | 0.0 | 5.4 |
| FokI | GGATG N9^ | 1.4 | 2.6 | 14.8 | 20.0 | 4.1 |
| Sau3AI | ^GATC | 0.0 | 0.0 | 0.0 | 0.0 | 0.0 |
|  |  |  |  |  |  |  |  |

Bold numbers indicate differences when compared with intervals in which strains are organized.

N – Any nucleotide; W – A or T.

* Data from REBASE [23]

Table S3. Chi-square independence test results according to MTase and geographic origin.

| MTase | Chi-square  (p value) | *Std. Residual** | | | | | Significant |
| --- | --- | --- | --- | --- | --- | --- | --- |
|  |  | MTase | Africa | Europe | America | Asia |  |
| M.AciI | 0.43559 | absence | -0.16 | -0.09 | 0.29 | 0.18 | No |
|  |  | presence | 0.65 | 0.38 | -1.21 | -1.21 |  |
|  |  |  |  |  |  |  |  |
| M.AseI | 0.03083 | absence | **2.13** | -1.53 | 0.70 | 0.55 | Yes |
|  |  | presence | -0.84 | 0.61 | -0.28 | -0.22 |  |
|  |  |  |  |  |  |  |  |
| M.BseRI | 0.66797 | absence | -0.72 | 0.72 | -0.61 | -0.37 | No |
|  |  | presence | 0.08 | -0.08 | 0.07 | 0.04 |  |
|  |  |  |  |  |  |  |  |
| M.BssHII | 0.79235 | absence | -0.59 | 0.59 | -0.49 | -0.30 | No |
|  |  | presence | 0.06 | -0.06 | 0.05 | 0.03 |  |
|  |  |  |  |  |  |  |  |
| M.BstUI | 0.00639 | absence | 0.33 | 0.39 | -0.16 | -1.87 | Yes |
|  |  | presence | -0.49 | -0.59 | 0.24 | **2.81** |  |
|  |  |  |  |  |  |  |  |
| M.DdeI | 0.40121 | absence | 0.93 | -0.39 | -0.08 | -0.17 | No |
|  |  | presence | -1.24 | 0.53 | 0.11 | 0.22 |  |
|  |  |  |  |  |  |  |  |
| DpnI | 0.35816 | absence | -1.31 | 0.93 | -0.20 | -0.67 | No |
|  |  | presence | 0.29 | -0.20 | 0.04 | 0.15 |  |
|  |  |  |  |  |  |  |  |
| M.DpnII | 0.11280 | absence | -1.38 | 1.01 | -0.30 | -0.71 | No |
|  |  | presence | 0.31 | -0.23 | 0.07 | 0.16 |  |
|  |  |  |  |  |  |  |  |
| M.DraI | <0.00001 | absence | 0.58 | 0.19 | -0.11 | -1.69 | Yes |
|  |  | presence | -1.85 | -0.61 | 0.36 | **5.36** |  |
|  |  |  |  |  |  |  |  |
| M.EagI | 0.22905 | absence | 1.58 | -1.01 | 0.73 | -0.43 | No |
|  |  | presence | -0.21 | 0.14 | -0.10 | 0.06 |  |
|  |  |  |  |  |  |  |  |
| M.FauI | 0.00403 | absence | 0.55 | 0.93 | -1.56 | **-2.04** | Yes |
|  |  | presence | -0.46 | -0.78 | 1.32 | 1.72 |  |
|  |  |  |  |  |  |  |  |
| M.Fnu4HI | 0.33023 | absence | 0.36 | 0.13 | -0.80 | 0.13 | No |
|  |  | presence | -0.65 | -0.23 | 1.45 | -0.23 |  |
|  |  |  |  |  |  |  |  |
| M.FokI | 0.00058 | absence | 0.09 | 0.33 | -0.57 | -0.51 | Yes |
|  |  | presence | -0.44 | -1.62 | **2.77** | **2.50** |  |
|  |  |  |  |  |  |  |  |
| M.HaeIII | 0.58574 | absence | -0.20 | -0.14 | 0.45 | 0.19 | No |
|  |  | presence | 0.48 | 0.32 | -1.06 | -0.43 |  |
|  |  |  |  |  |  |  |  |
| M.Hpy188I | 0.00177 | absence | 1.16 | 0.44 | -1.62 | -1.27 | Yes |
|  |  | presence | -1.47 | -0.55 | **2.05** | 1.61 |  |
|  |  |  |  |  |  |  |  |
| M.Hpy188III | 0.12633 | absence | -0.52 | 0.72 | 0.21 | -1.76 | No |
|  |  | presence | 0.35 | -0.48 | -0.14 | 1.18 |  |
|  |  |  |  |  |  |  |  |
| M.Hpy99I | 0.02544 | absence | 0.72 | 0.16 | -1.35 | 0.21 | Yes |
|  |  | presence | -1.21 | -0.27 | **2.29** | -0.36 |  |
|  |  |  |  |  |  |  |  |
| M.HpyCH4III | 0.00015 | absence | **-1.99** | 1.54 | **-2.21** | 1.63 | Yes |
|  |  | presence | 1.34 | -1.04 | 1.49 | -1.10 |  |
|  |  |  |  |  |  |  |  |

Table S3. (Continuation).

| MTase | chi-square (p-value) | | *Std. Residual** | | | | | Significant |
| --- | --- | --- | --- | --- | --- | --- | --- | --- |
|  |  |  | MTase | Africa | Europe | America | Asia |  |
| M.HpyCH4IV | | 0.14427 | absence | 1.21 | 0.16 | -1.19 | -1.02 | No |
|  | |  | presence | -0.73 | -0.10 | 0.72 | 0.62 |  |
|  | |  |  |  |  |  |  |  |
| M.HpyCH4V | | 0.35797 | absence | 1.20 | -0.81 | 0.42 | 0.06 | No |
|  | |  | presence | -0.77 | 0.52 | -0.27 | -0.04 |  |
|  | |  |  |  |  |  |  |  |
| M.MspI | | 0.03638 | absence | -1.77 | 1.30 | -0.25 | -1.10 | Yes |
|  | |  | presence | 1.12 | -0.82 | 0.16 | 0.69 |  |
|  | |  |  |  |  |  |  |  |
| M.NlaIII | | 0.18408 | absence | **2.00** | -0.81 | -0.35 | -0.21 | No |
|  | |  | presence | -0.13 | 0.05 | 0.02 | 0.01 |  |
|  | |  |  |  |  |  |  |  |
| M.Sau96I | | 0.06873 | absence | -0.85 | 0.16 | 0.29 | 0.56 | No |
|  | |  | presence | 1.93 | -0.37 | -0.67 | -1.28 |  |
|  | |  |  |  |  |  |  |  |
| M.ScrFI | | 0.99456 | absence | -0.04 | 0.05 | -0.06 | 0.00 | No |
|  | |  | presence | 0.11 | -0.14 | 0.19 | 0.00 |  |
|  | |  |  |  |  |  |  |  |
| M.TaqI | | 0.04391 | absence | -1.74 | 1.51 | -1.31 | -0.24 | Yes |
|  | |  | presence | 0.66 | -0.58 | 0.50 | 0.09 |  |

* Standardized residuals determined for absence or presence of expression of each MTase. When the association is strong (>1.96 or <-1.96) the values are in bold. An MTase is associated with an origin when the value of Std. residual to absence of expression is negative. or when this value is positive in case of presence of expression. The absence of expression is associated to an origin in the opposite situation.

**Table S4. Fischer test results between geographic origins and significant MTases determined by chi-square test.**

| MTase | Fischer test (p-value) | Significant |
| --- | --- | --- |
| M.AseI | 0.02324 | Yes |
| M.BstUI | 0.00978 | Yes |
| M.DraI | 0.00002 | Yes |
| M.FauI | 0.00203 | Yes |
| M.FokI | 0.00192 | Yes |
| M.Hpy188I | 0.00172 | Yes |
| M.Hpy99I | 0.03080 | Yes |
| M.HpyCH4III | 0.00006 | Yes |
| M.MspI | 0.03453 | Yes |
| M.TaqI | 0.03408 | Yes |

Table S5. Variables of the logistic regression model (Europe or non-Europe).

| MTase^a)^ | B^b)^ | S.E..^c)^ | Wald^d)^ | df^e)^ | Sig.^d)^ | Exp(B) (OR)^f)^ | EXP(B) 95.0% CI ^g)^ | |
| --- | --- | --- | --- | --- | --- | --- | --- | --- |
|  |  |  |  |  |  |  | Inferior | Superior |
| M.AseI | 0.85 | 0.43 | 3.80 | 1 | **0.05125** | 2.33 | 1.00 | 5.46 |
| M.FokI | -2.10 | 0.89 | 5.59 | 1 | **0.01808** | 0.12 | 0.02 | 0.70 |
| M.MspI | -0.80 | 0.37 | 4.58 | 1 | **0.03235** | 0.45 | 0.22 | 0.94 |
| M.Hpy188I | -0.16 | 0.32 | 0.25 | 1 | 0.61393 | 0.85 | 0.45 | 1.60 |
| M.Hpy99I | -0.24 | 0.35 | 0.48 | 1 | 0.48837 | 0.79 | 0.40 | 1.56 |
| M.HpyCH4III | -0.88 | 0.38 | 5.23 | 1 | **0.02219** | 0.41 | 0.20 | 0.88 |
| M.DraI | -0.42 | 0.56 | 0.56 | 1 | 0.45344 | 0.66 | 0.22 | 1.98 |
| M.BstUI | 0.18 | 0.34 | 0.28 | 1 | 0.59401 | 1.20 | 0.61 | 2.34 |
| M.FauI | -0.35 | 0.32 | 1.18 | 1 | 0.27663 | 0.70 | 0.37 | 1.33 |
| M.TaqI | -0.77 | 0.62 | 1.57 | 1 | 0.21025 | 0.46 | 0.14 | 1.55 |
| Constant | 2.29 | 0.80 | 8.25 | 1 | 0.00407 | 9.91 |  |  |

a) Simultaneous introduction of all MTases in the model (Enter method);

b) Values for the logistic regression equation;

c) Standard errors associated with the coefficients;

d) Wald statistic (chi-square) and test significance. Significant values (<0.05) are in bold;

e) Degrees of freedom for each of the tests of the coefficients;

f) Odds ratios (OR) for the predictors, which are the exponentiation of the coefficients.

g) OR 95% confidence interval.

**Table S6. Variables of the logistic regression model (African or non-African).**

| MTase ^a)^ | B^b)^ | S.E.^c)^ | | Wald^d)^ | | df^e)^ | Sig.^d)^ | Exp(B) (OR)^f)^ | | EXP(B) 95.0% C. I. ^g)^ | |
| --- | --- | --- | --- | --- | --- | --- | --- | --- | --- | --- | --- |
|  |  |  |  |  |  |  |  |  |  | Inferior | Superior |
| M.AseI | -1.31 | | 0.52 | | 6.25 | 1 | **0.01244** | | 0.27 | 0.10 | 0.75 |
| M.FokI | -1.17 | | 1.23 | | 0.90 | 1 | 0.34375 | | 0.31 | 0.03 | 3.49 |
| M.MspI | 1.49 | | 0.57 | | 6.87 | 1 | **0.00875** | | 4.42 | 1.46 | 13.43 |
| M.Hpy188I | -1.04 | | 0.46 | | 5.14 | 1 | **0.02341** | | 0.35 | 0.14 | 0.87 |
| M.Hpy99I | -1.05 | | 0.53 | | 3.93 | 1 | **0.04749** | | 0.35 | 0.12 | 0.99 |
| M.HpyCH4III | 1.49 | | 0.57 | | 6.92 | 1 | **0.00850** | | 4.44 | 1.46 | 13.47 |
| M.DraI | -19.59 | | 7909.31 | | 0.00 | 1 | 0.99802 | | 0.00 | 0.00 | . |
| M.BstUI | -0.65 | | 0.47 | | 1.94 | 1 | 0.16376 | | 0.52 | 0.21 | 1.30 |
| M.FauI | -0.33 | | 0.41 | | 0.66 | 1 | 0.41634 | | 0.72 | 0.32 | 1.60 |
| M.TaqI | 1.26 | | 1.09 | | 1.32 | 1 | 0.24990 | | 3.52 | 0.41 | 29.92 |
| Constant | -2.93 | | 1.27 | | 5.27 | 1 | 0.02166 | | 0.05 |  |  |

a) Simultaneous introduction of all MTases in the model (Enter method);

b) Values for the logistic regression equation;

c) Standard errors associated with the coefficients;

d) Wald statistic (chi-square) and test significance. Significant values (<0.05) are in bold;

e) Degrees of freedom for each of the tests of the coefficients;

f) Odds ratios (OR) for the predictors, which are the exponentiation of the coefficients.

g) OR 95% confidence interval.

Table S7. Variables in the multinomial logistic regression equation with 8 independent variables (reference: Africa).

| Origin * | MTase ^a)^ | B^b)^ | S.E.^c)^ | Wald^d)^ | df^e)^ | Sig.^d)^ | Exp(B) / OR^f)^ | Exp(B) 95% C. I. ^g)^ | |
| --- | --- | --- | --- | --- | --- | --- | --- | --- | --- |
|  |  |  |  |  |  |  |  | Inferior | Superior |
| European | Intercept | 21.38 | 1.10 | 374.84 | 1 | 0.00000 |  |  |  |
|  | [M.AseI=0] | -1.38 | 0.54 | 6.56 | 1 | **0.01044** | 0.25 | 0.09 | 0.72 |
|  | [M.MspI=0] | 1.51 | 0.57 | 7.08 | 1 | **0.00779** | 4.51 | 1.49 | 13.67 |
|  | [M.Hpy188I=0] | -0.80 | 0.46 | 3.04 | 1 | 0.08102 | 0.45 | 0.18 | 1.10 |
|  | [M.Hpy99I=0] | -0.78 | 0.53 | 2.18 | 1 | 0.13941 | 0.46 | 0.16 | 1.29 |
|  | [M.HpyCH4III=0] | 1.47 | 0.55 | 7.00 | 1 | **0.00815** | 4.34 | 1.46 | 12.87 |
|  | [M.DraI=0] | -19.03 | 0.89 | 456.07 | 1 | **0.00000** | 0.00 | 0.00 | 0.00 |
|  | [M.BstUI=0] | -0.62 | 0.47 | 1.70 | 1 | 0.19211 | 0.54 | 0.21 | 1.36 |
|  | [M.TaqI=0] | 1.23 | 1.08 | 1.29 | 1 | 0.25684 | 3.42 | 0.41 | 28.54 |
| American | Intercept | 21.39 | 1.29 | 275.27 | 1 | 0.00000 |  |  |  |
|  | [M.AseI=0] | -0.65 | 0.71 | 0.83 | 1 | 0.36251 | 0.52 | 0.13 | 2.11 |
|  | [M.MspI=0] | 1.01 | 0.72 | 1.99 | 1 | 0.15830 | 2.75 | 0.67 | 11.25 |
|  | [M.Hpy188I=0] | -1.80 | 0.59 | 9.20 | 1 | **0.00242** | 0.17 | 0.05 | 0.53 |
|  | [M.Hpy99I=0] | -1.84 | 0.64 | 8.26 | 1 | **0.00405** | 0.16 | 0.05 | 0.56 |
|  | [M.HpyCH4III=0] | -0.19 | 0.92 | 0.04 | 1 | 0.83435 | 0.83 | 0.14 | 5.00 |
|  | [M.DraI=0] | -19.34 | 1.08 | 321.60 | 1 | **0.00000** | 0.00 | 0.00 | 0.00 |
|  | [M.BstUI=0] | -0.30 | 0.62 | 0.24 | 1 | 0.62503 | 0.74 | 0.22 | 2.49 |
|  | [M.TaqI=0] | 0.12 | 1.51 | 0.01 | 1 | 0.93642 | 1.13 | 0.06 | 21.87 |
| Asian | Intercept | 21.91 | 1.14 | 370.40 | 1 | 0.00000 |  |  |  |
|  | [M.AseI=0] | -1.35 | 1.28 | 1.11 | 1 | 0.29270 | 0.26 | 0.02 | 3.20 |
|  | [M.MspI=0] | 0.13 | 1.37 | 0.01 | 1 | 0.92546 | 1.14 | 0.08 | 16.82 |
|  | [M.Hpy188I=0] | -1.63 | 0.95 | 2.98 | 1 | 0.08419 | 0.20 | 0.03 | 1.25 |
|  | [M.Hpy99I=0] | -0.88 | 1.10 | 0.64 | 1 | 0.42307 | 0.42 | 0.05 | 3.57 |
|  | [M.HpyCH4III=0] | 2.83 | 1.01 | 7.80 | 1 | **0.00523** | 16.98 | 2.33 | 123.98 |
|  | [M.DraI=0] | -21.73 | 0.00 | . | 1 | . | 0.00 | 0.00 | 0.00 |
|  | [M.BstUI=0] | -2.72 | 1.01 | 7.31 | 1 | **0.00687** | 0.07 | 0.01 | 0.47 |
|  | [M.TaqI=0] | -0.21 | 1.82 | 0.01 | 1 | 0.90704 | 0.81 | 0.02 | 28.89 |

* Reference category: Africa;

a) Simultaneous introduction of all MTases in the model (Enter method);

b) Values for the logistic regression equation;

c) Standard errors associated with the coefficients;

d) Wald statistic (chi-square) and test significance. Significant values (<0.05) are in bold;

e) Degrees of freedom for each of the tests of the coefficients;

f) Odds ratios (OR) for the predictors, which are the exponentiation of the coefficients.

g) OR 95% confidence interval.

Table S8. Variables in the multinomial logistic regression equation with 8 independent variables (reference: Europe).

| Origin* | MTase^a)^ | B^b)^ | S.E.^c)^ | Wald^d)^ | gl^e)^ | Sig.^d)^ | Exp(B)  /OR^f)^ | Exp(B) 95% C. I.^g)^ | |
| --- | --- | --- | --- | --- | --- | --- | --- | --- | --- |
|  |  |  |  |  |  |  |  | Inferior | Superior |
| African | Intercept | -19.38 | 0.68 | 816.80 | 1 | 1E-179 |  |  |  |
|  | [M.AseI=0] | 1.38 | 0.54 | 6.56 | 1 | **0.01044** | 3.98 | 1.38 | 11.45 |
|  | [M.MspI=0] | -1.51 | 0.57 | 7.08 | 1 | **0.00779** | 0.22 | 0.07 | 0.67 |
|  | [M.Hpy188I=0] | 0.80 | 0.46 | 3.04 | 1 | 0.08102 | 2.23 | 0.91 | 5.49 |
|  | [M.Hpy99I=0] | 0.78 | 0.53 | 2.18 | 1 | 0.13941 | 2.19 | 0.77 | 6.20 |
|  | [M.HpyCH4III=0] | -1.47 | 0.55 | 7.00 | 1 | **0.00815** | 0.23 | 0.08 | 0.68 |
|  | [M.DraI=0] | 17.03 | 0.00 | . | 1 | . | 2.48E+07 | 2.48E+07 | 2.48E+07 |
|  | [M.BstUI=0] | 0.62 | 0.47 | 1.70 | 1 | 0.19211 | 1.85 | 0.73 | 4.68 |
|  | [M.TaqI=0] | -1.23 | 1.08 | 1.29 | 1 | 0.25684 | 0.29 | 0.04 | 2.45 |
| American | Intercept | 0.01 | 0.88 | 0.00 | 1 | 0.99043 |  |  |  |
|  | [M.AseI=0] | 0.73 | 0.63 | 1.37 | 1 | 0.24171 | 2.08 | 0.61 | 7.09 |
|  | [M.MspI=0] | -0.49 | 0.52 | 0.89 | 1 | 0.34442 | 0.61 | 0.22 | 1.70 |
|  | [M.Hpy188I=0] | -1.00 | 0.47 | 4.53 | 1 | **0.03327** | 0.37 | 0.15 | 0.92 |
|  | [M.Hpy99I=0] | -1.06 | 0.48 | 4.93 | 1 | **0.02642** | 0.35 | 0.14 | 0.88 |
|  | [M.HpyCH4III=0] | -1.66 | 0.78 | 4.55 | 1 | **0.03283** | 0.19 | 0.04 | 0.87 |
|  | [M.DraI=0] | -0.31 | 0.79 | 0.16 | 1 | 0.69260 | 0.73 | 0.16 | 3.42 |
|  | [M.BstUI=0] | 0.31 | 0.50 | 0.40 | 1 | 0.52805 | 1.37 | 0.52 | 3.63 |
|  | [M.TaqI=0] | -1.11 | 1.11 | 0.99 | 1 | 0.32023 | 0.33 | 0.04 | 2.93 |
| Asian | Intercept | 0.53 | 1.12 | 0.22 | 1 | 0.63557 |  |  |  |
|  | [M.AseI=0] | 0.03 | 1.21 | 0.00 | 1 | 0.97951 | 1.03 | 0.10 | 11.06 |
|  | [M.MspI=0] | -1.38 | 1.26 | 1.19 | 1 | 0.27497 | 0.25 | 0.02 | 2.99 |
|  | [M.Hpy188I=0] | -0.83 | 0.85 | 0.95 | 1 | 0.32904 | 0.44 | 0.08 | 2.31 |
|  | [M.Hpy99I=0] | -0.09 | 0.99 | 0.01 | 1 | 0.92382 | 0.91 | 0.13 | 6.35 |
|  | [M.HpyCH4III=0] | 1.36 | 0.87 | 2.45 | 1 | 0.11762 | 3.91 | 0.71 | 21.63 |
|  | [M.DraI=0] | -2.70 | 0.89 | 9.20 | 1 | **0.00242** | 0.07 | 0.01 | 0.38 |
|  | [M.BstUI=0] | -2.10 | 0.91 | 5.31 | 1 | **0.02125** | 0.12 | 0.02 | 0.73 |
|  | [M.TaqI=0] | -1.44 | 1.48 | 0.95 | 1 | 0.33051 | 0.24 | 0.01 | 4.31 |

* Reference category: Europe;

a) Simultaneous introduction of all MTases in the model (Enter method);

b) Values for the logistic regression equation;

c) Standard errors associated with the coefficients;

d) Wald statistic (chi-square) and test significance. Significant values (<0.05) are in bold;

e) Degrees of freedom for each of the tests of the coefficients;

f) Odds ratios (OR) for the predictors, which are the exponentiation of the coefficients.

g) OR 95% confidence interval.
